# Molecular Detection of* Theileria* spp. in Livestock on Five Caribbean Islands

**DOI:** 10.1155/2015/624728

**Published:** 2015-12-09

**Authors:** Jilei Zhang, Patrick Kelly, Jing Li, Chuanling Xu, Chengming Wang

**Affiliations:** ^1^Jiangsu Co-Innovation Center for Prevention and Control of Important Animal Infectious Diseases and Zoonoses, Yangzhou University College of Veterinary Medicine, Yangzhou, Jiangsu 225009, China; ^2^Ross University School of Veterinary Medicine, Basseterre, Saint Kitts and Nevis

## Abstract

*Theileria* spp. are tick-transmitted, intracellular apicomplexan protozoan parasites infecting a wide range of animals. As there is very limited information on the prevalence of* Theileria* spp. in the Caribbean we used the recently described genus-specific pan-*Theileria* FRET-qPCR to identify infected animals in the region and a standard 18S rRNA gene PCR and sequencing to determine the species involved. We found* Theileria* spp. in 9% of the convenience samples of animals (*n* = 752) studied from five Caribbean islands. Donkeys (20.0%: 5/25) were most commonly infected, followed by sheep (17.4%, 25/144), cattle (6.8%; 22/325), goats (5.0%; 12/238), and horses (5.0%; 1/20). Six species of* Theileria* were identified:* T*.* equi* (donkeys, cattle, goats, and sheep),* Theileria* sp. OT3 (sheep and goats),* Theileria *sp. NG-2013a (cattle),* Theileria* sp. YW-2014 (donkeys),* Theileria* sp. B15a (goats), and* Babesia vulpes* or a closely related organism (sheep and goats). Only* T. equi* has been previously reported in the Caribbean. Our findings expand the known host ranges of* Theileria* spp. and the known distribution of the organisms around the world.

## 1. Background

*Theileria* spp. are tick-transmitted, intracellular apicomplexan protozoan parasites infecting leukocytes and erythrocytes of a wide range of animals [1, 2]. The organisms have been described in all livestock species and can cause significant economic losses to farmers. They are transmitted by a variety of ixodid ticks of the genera* Rhipicephalus*,* Hyalomma*,* Amblyomma,* and* Haemaphysalis* [3]. Infections with some* Theileria* spp. can result in fever, anemia, hemoglobinuria, and death in severe cases, but many species are benign and cause minor or no signs. Animals that recover from acute or primary infections usually remain persistently infected and may act as reservoirs for tick vectors [4, 5]. Infected animals are found particularly in tropical and subtropical regions in Africa, the Middle East, Southern Europe, and Asia [6–11].

There is little information on infectious agents in livestock in the Caribbean although animal production is an important source of income for many people in the region. In the case of* Theileria* spp., morphological and serological evidence has been presented that* T. mutans* and* T. velifera*, both benign species transmitted by* Amblyomma* spp., occur in cattle on Guadeloupe [12]. Also, an organism with the morphology of* T. mutans* was seen in a blood smear from a bovine on Martinique [13]. In Trinidad,* T. equi* (previously* Babesia equi*) has been demonstrated in horses with a specific nested 18S rRNA PCR [14, 15] and a serosurvey has provided supporting evidence for its presence [16].

While there are many tests to detect* Theileria* spp. in animals, their specificity varies, as does their usefulness in finding the full spectrum of organisms present in an area. Microscopic detection of parasites can be difficult with low parasitemia and does not readily allow differentiation of species [2]. Serological studies, although sensitive and relatively easy to perform, are not specific as there is cross-reactivity between* Theileria* spp. [17]. Although molecular techniques have been described, many are for specific species which limits their usefulness in surveys. Reverse line blotting (RLB) assays enable the simultaneous identification of multiple species [18, 19], but they are cumbersome and time demanding to perform and identifying stringent species-specific oligonucleotide sequences can be challenging [20]. Recently, a sensitive genus-specific pan-*Theileria* FRET-qPCR has been described that detects the recognized* Theileria* spp. of domestic animals in a single reaction (Table 1) [21]. To provide further data on* Theileria* spp. in the Caribbean, we used the pan-*Theileria* FRET-qPCR to screen livestock from five islands for evidence of infection. Further, we used a standard 18S rRNA PCR and gene sequencing on positive reactors to identify the* Theileria* spp. involved. The results of this survey are described below.

## 2. Materials and Methods

### 2.1. Samples Collection

Jugular venipuncture was used to collect blood in EDTA from convenience samples of apparently healthy livestock (cattle, goats, sheep, donkeys, and horses) on five Caribbean islands [22]. This study was reviewed and approved by the Institutional Animal Care and Use Committee of the Ross University School of Veterinary Medicine (RUSVM), St. Kitts. Owners of animals gave permission for the blood samples to be collected.

### 2.2. DNA Extraction

The DNA was extracted from aliquots (200 *μ*L) of the whole blood samples with the QIAamp DNA Blood Mini Kit (QIAGEN, Valencia, CA, USA) according to the manufacturer's instructions. The DNAs were eluted into 200 *μ*L Buffer AE and couriered to Yangzhou University College of Veterinary Medicine, China, at room temperature where they were frozen at −80°C until PCRs were performed.

### 2.3. PCRs for* Theileria* Detection and Species Determination

**Figure 1:**
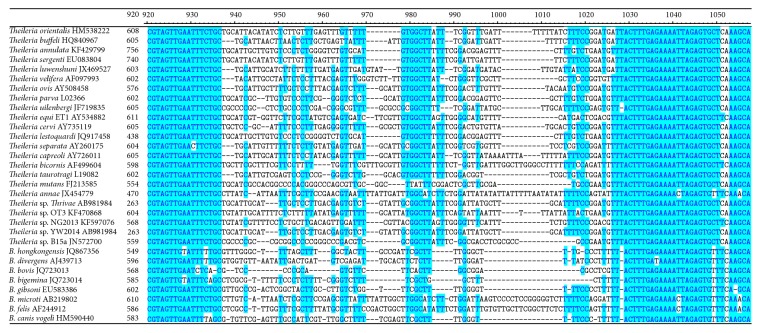
Alignment of the sequences in the polymorphic region of the 18S RNA gene of* Theileria* spp. and related organisms that was targeted in the standard PCR used in the study. Nucleotides that are identical for all species are highlighted in blue while those that vary between species and can be used for differentiation are not highlighted.

All the PCRs were performed on a Roche Light-Cycler 480-II platform with the HMBS gene as an endogenous control [23]. Samples found positive in the pan-*Theileria* FRET-qPCR were tested in a conventional PCR with primers targeting a highly polymorphic 584–610-nucleotide region of the 18S rRNA gene of* Theileria* spp. (Table 2, Figure 1) [21]. The amplicons from positive conventional PCRs were sequenced directly with forward and reverse primers to determine the* Theileria* spp. present (BGI, Shanghai, China) [21] as has been done with 18S rRNA sequences in a number of previous studies [11, 14, 17, 21, 24].

## 3. Results and Discussion

**Figure 2:**
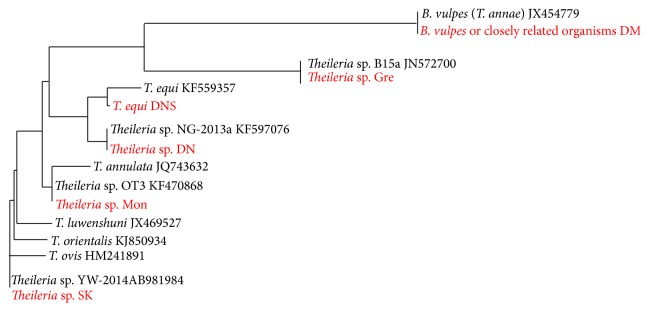
Phylogenetic tree of sequences identified in this study and closely related* Theileria* spp. Sequences from GenBank have gene accession numbers in black font; those from this study are in red font.* B. vulpes* or closely related organism DM indicates the organism detected on Dominica and Montserrat;* Theileria* sp. Gre indicates the* Theileria* sp. from Grenada;* T. equi* DNS indicates the sequence of* T. equi* from Dominica, Nevis, and St. Kitts;* Theileria* sp. Mon indicates the* Theileria* sp. detected from Montserrat; and* Theileria* sp. SK is the sequence of* Theileria* sp. from St. Kitts.

The sensitive and specific pan-*Theileria* FRET-qPCR [21] we used in our study demonstrated that a substantial proportion of livestock (8.6%; 65/752) on the five Caribbean islands we studied were infected with* Theileria* (Table 3). Each island had positive animals and each livestock species we studied was found to be infected with* Theileria* spp. Sequencing of representative samples of positive 18S rRNA PCR amplicons we obtained (*n* = 43) showed that there was one recognized* Theileria* spp. (*T. equi*) present in the Caribbean, along with five less well characterized* Theileria* sp. (Tables 3 and 4, Figure 2). The average copy number of 18S rRNA per mL whole blood was relatively low at 116.6 ± 440.8, indicating that the animals we studied were chronically infected.

*Theileria equi* and the* Theileria* sp. YW-2014 were the species we identified in equids.* Theileria* sp. YW-2014 has been described in a Sika deer (*Cervus nippon*) from Japan but there is little sequence data on the organism with only a 552 bp sequence of the 18S rRNA gene reported in GenBank (AB981984). On the other hand,* T. equi* is a well-recognized cause of equine piroplasmosis [25], an important disease of horses which has been recognized and studied in the Caribbean [14–16]. The organism has also been found in dogs in Spain [26], South Africa [27], and Nigeria [28] with some having clinical signs that responded to appropriate treatment [29]. Our finding that* T. equi* also occurs in domestic ruminants further expands the host range of the organism. The significance, extent, and consequences of infections with* T. equi* in domestic ruminants require further investigation.

Numerous species of* Amblyomma*,* Dermacentor, Hyalomma*,* Ixodes,* and* Rhipicephalus* are confirmed or suspected vectors of* T. equi* [29]. Of these, only* A. cajennense* [30],* R. microplus* [30],* R. sanguineus* [31], and* R. turanicus* (unpublished observation) occur in the Caribbean. There is conflicting data that* Amblyomma cajennense* is a competent vector of* T. equi* [32] but the tick is localized to Jamaica, Trinidad, and Cuba in the Caribbean [30] and it appears, then, not to be the vector of* T. equi* we found on Nevis, St. Kitts, and Dominica.* Dermacentor nitens*, the tropical horse tick, is very common in the Caribbean and the tropical Americas [33]. Although there is no data on the competence of* D. nitens* as a vector of* T. equi* and there is contradictory epidemiological evidence [32], PCR positive* D. nitens* have been found [32] and Asgarali et al. [16] have suggested that this tick is a vector in Trinidad. They also suggested that* R. microplus* [16], which is very common on cattle throughout the Caribbean, might also be a vector. While there is some evidence that* R. microplus* is a competent vector [34] and our PCR identified* T. equi* in cattle on two islands, it seems unlikely that* R. microplus* is an important natural vector as it is a one host species and transovarial transmission has not been demonstrated [32]. Although* R. sanguineus* and* R. turanicus* have been implicated as vectors of* T. equi*, more recent studies have failed to confirm their role [29]. Further studies are needed to establish the epidemiology of* T. equi* and its vectors in the Caribbean and the neighboring Americas [32].

*Theileria* sp. OT3 was first described in sheep, deer, and chamois in Spain [35–37] and later in sheep in Italy [38], China [39], and Turkey [40]. Its pathogenicity and vectors have yet to be determined. Ours is the first report of the organism in the Caribbean and also the first report of the* Theileria* sp. OT3 in goats which further demonstrates that the organism has a wide distribution and host range. Although we used only convenience samples which were not representative of the islands, it is of note that* Theileria* sp. OT3 was the most prevalent species we detected in ruminants. Similar high prevalences of infections have also been reported in the other countries where the organism has been described. The sequences of our* Theileria* sp. OT3 were identical to one another and to that of an isolate from China (KF470868) [39]. A phylogenetic relationship tree established for the Chinese isolate showed that the* Theileria* sp. OT3 forms a separated cluster and that the organism is closely related to* T. uilenbergi*,* T. luwenshuni,* and* T. ovis*. Although the organism has only been found in apparently healthy animals, studies on its pathogenicity seem indicated as, with its high prevalence and wide distribution, it might be causing substantial economic losses for livestock farmers.

*Theileria mutans* and* T. velifera* are African species that have been reported on Guadeloupe [12] and appear most likely to have been imported with cattle from West Africa in the 18th Century. This might also have been the case with the other African* Theileria* sp. we identified. Seven of the* Theileria* we found had an identical sequence to that of* Theileria* sp. NG-2013a (KF597076) described from waterbuck (*Kobus defassa*) in Kenya [41]. This organism has been found to cluster with* T. equi* but might represent a novel taxon. Its vectors and pathogenicity are unknown. One goat we studied had a* Theileria* spp. with a sequence identical to that of* Theileria* sp. B15a (JN572700) from an African buffalo (*Syncerus caffer*) in South Africa [42]. This organism is close to* T. mutans* which is widespread in Africa where it is transmitted by* Amblyomma* spp. and causes benign theileriosis. Previously* T. mutans* has been reported on Guadeloupe based on serological test results [12] and, since we found no confirmatory molecular evidence for the presence of* T. mutans*, it might be that the cross-reacting antibodies detected in Guadeloupe were against this closely related* Theileria* sp. B15.

The only non-*Theileria* sp., the pan-*Theileria* FRET-qPCR identified in our study, was one that appeared to be* Babesia vulpes* [43] or a closely related species. This organism was previously known as “*T. annae*” or the “*Babesia microti*-like organism” but was reclassified based on 18S rRNA and tubulin-beta gene sequence data [43]. The sequences of the short amplicons we obtained in the pan-*Theileria* FRET-qPCR were identical to those of* B. vulpes* (JX454779; KF773740) and had one mismatch with* B. microti* (AB219802, HQ629933, and LC005772). We were unable, however, to obtain longer amplicons with the standard PCR, probably because there were only very low copy numbers present in the positive animals. We were, then, unable to use this additional sequencing data to confirm that the organism we detected was* B. vulpes* or determine if it was a closely related species or strain. The pan-*Theileria* FRET-qPCR we used in our study was designed to detect seventeen recognized* Theileria* spp., which did not include “*T. annae*” or* B. vulpes* (Table 1) [21]. These seventeen recognized* Theileria* spp. differed from one another by only a maximum of 4 nucleotides in the regions of the primers and the probes used in the pan-*Theileria* FRET-qPCR. It is of note, then, that these primers and probes enabled the multiplication and detection of* B. vulpes*, or a closely related species, with 8 nucleotide differences and it therefore appears that the pan-*Theileria* FRET-qPCR might not be as genus specific as first thought. Further work is currently underway in our laboratory to more clearly characterize the* B. vulpes* or closely related organism found in the Caribbean.

## 4. Conclusions

Our study has confirmed the sensitivity of the pan-*Theileria* PCR in the rapid detection of a wide range of* Theileria* spp. but has also shown it might detect* B. vulpes* or closely related organisms. We found livestock infected with* Theileria* spp. on each of the five islands we studied. While we could not confirm previous reports of* T. mutans* and* T. velifera* in cattle, we found that one recognized species,* T. equi*, four poorly characterized* Theileria* spp., and* B. vulpes* or a closely related organism are present in the region. Further studies are indicated to more precisely determine the phylogenetic relationships of these organisms in the Caribbean with closely related organisms from other parts of the world. Also, the prevalences of infections on the different islands should be determined as well as the impact these poorly characterized organisms might have on livestock production, both in the Caribbean and around the world where they are found.

## Figures and Tables

**Table 1:** Alignment of the nucleotides used in the primers and probes of the pan-*Theileria* FRET-qPCR used in this study.

	Forward primer	LCRed-640	6-FAM	Reverse primer
	T AGTGACAAGAAATAACAATACGGGGC- -TT	GTCTTGTAATTGGAATGATGGGAATT	AAACCTCTTCCAGAGTATCAATTGG	AGTTAAAAAGCTCGTAGTTGAATTTCTGCTG
*T. orientalis *	...........................- -..	..........................	.........................	...............................
*T. buffeli *	...........................- -.C	..........................	.........................	...............................
*T. annulata *	........................... - - ..	..........................	.........................	...............................
*T. sergenti *	........................... - - ..	..........................	.........................	...............................
*T. luwenshuni *	........................... - - ..	..........................	.........................	...............................
*T. velifera *	........................... - - ..	.C........................	.........................	..............................A
*T. ovis *	........................... - - ..	..........................	.........................	...............................
*T. parva *	........................... - - ..	..........................	.........................	...............................
*T. uilenbergi *	........................... - - ..	..........................	.........................	.............................C.
*T. lestoquardi *	........................... - - ..	..........................	.........................	...............................
*T. equi *	......................A.... - - ..	..........................	.....C...................	...............................
*T. separata *	........................... - - ..	..........................	.........................	......................C........
*T. capreoli *	........................... - - ..	..........................	.........................	...............................
*T. bicornis *	........................... - - ..	.....C....................	.........................	...............................
*T. taurotragi *	........................... - - ..	..........................	.........................	...............................
*T. mutans *	........................... - - .C	.C.......................C	.....C...................	...............................
*Theileria* sp. OT3	........................... - - ..	..........................	.........................	...............................
*Theileria* sp. NG	........................... - - ..	..........................	.........................	...............................
*Theileria* sp. YW	........................... - - ..	..........................	.........................	...............................
*Theileria* sp. B15	........................... - - .C	.C......................CC	.....C...................	.............................C.
*B. vulpes *	......................A.... - - ..	.........................C	.....CT.C................	......G......................CT
*B. hongkongensis *	......................A.... - - AA	.....................TG.C.	.....CTCA........G.......	....................T....T...GT
*B. divergens *	......................A.... - - AA	.....................TG.CC	.....CTCA........A.......	.........................T...GT
*B. bovis *	...............C........... - - .A	.CTC.........C...GG..CG.CC	TC...CTCG..C.....C.C.....	........................C.A-.GT
*B. bigemina *	......................A.... - - ..	.....................TG..G	.C.A.CTCA........C.......	....G...............T.....A..CT
*B. gibsoni *	......................A.... - - AA	.....................TG.CG	...ATCTCA........A.......	.A...........................GT
*B. microti *	......................A.... - - ..	.........................C	.....CT.C................	......G......................CT
*B. felis *	......................A.... - - ..	......................G.CC	.....CT.C................	......G......................CT
*B. canis *	......................A.... - - .A	.....................TG.C.	.....CTCA........G.......	.........................TA..GT
*C. felis *	.......................A... - - ..	..................C..A....	..G.TCT....G.............	...............................
*H. americanum *	......................AA...AA..	.CT................A.A....	....ACT..TT.A............	............................T.A
*T. gondii *	...................C..T..AAAT..	T...A..G...........A.....C	.....C...T.......A.......	....................G..........

Primers and probes are shown at the head of the table. Dots indicate nucleotides identical to primers and probes, and dashes denote absence of the nucleotide. The upstream primer is used as the demonstrated sequences without gaps while the two probes and downstream primer are used as antisense oligonucleotides. The designed oligonucleotides show minimum mismatching with *Theileria* spp. The 6-FAM label is directly attached to the 3-terminal nucleotide of the fluorescein probe, and the LCRed-640 fluorescein label is added via a linker to the 5′-end of the LCRed-640 probe. The 18S rRNA sequences for the available recognized *Theileria* spp. on GenBank and other closely related protozoan species were obtained from GenBank: *T. orientalis* (HM538222), *T. buffeli* (HQ840967), *T. annulata* (KF429799), *T. sergenti* (EU083804), *T. luwenshuni* (JX469527), *T. velifera* (AF097993), *T. ovis* (AY508458), *T. parva* (L02366), *T. uilenbergi* (JF719835), *T. equi* (AY534882), *T. lestoquardi* (JQ917458), *T. separata* (AY260175), *T. capreoli* (AY726011), *T. bicornis* (AF499604), *T. taurotragi* (L19082), *T. mutans* (FJ213585), *Babesia vulpes *(JX454779), *Theileria* sp. OT3 (KF470868), *Theileria* sp. NG-2013a (KF597076), *Theileria* sp. YW-2014 (AB981984), *Theileria* sp. B15a (JN572700); *B. hongkongensis* (JQ867356), *B. divergens* (AJ439713), *B. bovis* (JQ723013), *B. bigemia* (JQ723014), *B. gibsoni* (EU583386), *B. microti* (AB219802), *B. felis* (AF244912), *B. canis* (HM590440), *Hepatozoon americanum* (AF176836), *Cytauxzoon felis* (AY679105), and *Toxoplasma gondii* (L37415).

**Table 2:** Primers and probes used in this study.

PCR	Primer/probe	Nucleotides sequence	Amplicon
FRET-qPCR	Forward Reverse 6-FAM LCRed-640	5′-TAGTGACAAGAAATAACAATACGGGGCTT-3′ 5′-CAGCAGAAATTCAACTACGAGCTTTTTAACT-3′ 5′-CCAATTGATACTCTGGAAGAGGTTT-(6-FAM)-3′ 5′-(LCRed640)-AATTCCCATCATTCCAATTACAAGAC-Phosphate-3′	178 bp

Conventional PCR	Upstream Downstream	5′-CCTGAGAAACGGCTACCACATCT-3′ 5′-GGACTACGACGGTATCTGATCG-3′	*T. orientalis* 593 bp; *T. buffeli* 591 bp; *T. annulata* 591 bp; *T. sergenti* 591 bp; *T. luwenshuni* 594 bp; *T. velifera* 592 bp; *T. ovis* 595 bp; *T. parva* 592 bp; *T. uilenbergi* 592 bp; *T. equi* 596 bp; *T. cervi* 595 bp; *T. lestoquardi* 591 bp; *T. separata* 593 bp; *T. capreoli* 599 bp; *T. bicornis* 610 bp; *T. taurotragi* 587 bp; *T. mutans* 585 bp; *B. vulpes *609 bp; *Theileria* sp. OT3 600 bp; *Theileria* sp. NG 597 bp; *Theileria* sp. YW 593 bp; *Theileria* sp. B15 584 bp

**Table 3:** Prevalence of *Theileria* spp. in livestock from five Caribbean islands.

	Bovine	Goat	Sheep	Donkey	Horse	Total	*Theileria* spp.
Dominica	3/77 (3.9%)	0/70 (0.0%)	1/15 (6.7%)	N/A^*∗*^	N/A	4/162 (2.5%)	*T. equi, Theileria* sp. NG-2013a, *B. vulpes, *or closely related organism

Grenada	N/A	2/31 (6.5%)	N/A	N/A	N/A	2/31 (6.5%)	*Theileria* sp. B15a

Montserrat	0/12 (0.0%)	8/19 (42.1%)	24/62 (38.7%)	N/A	N/A	32/93 (34.4%)	*Theileria* sp. OT3, *B. vulpes, *or closely related organism

Nevis	19/43 (44.2%)	2/114 (1.8%)	0/41 (0.0%)	N/A	N/A	21/198 (10.6%)	*T. equi*, *Theileria* sp. NG-2013a

St. Kitts	0/193 (0.0%)	0/4 (0.0%)	0/26 (0.0%)	5/25 (20.0%)	1/20 (5.0%)	6/268 (2.2%)	*T. equi*, *Theileria* sp. YW-2014

Total	22/325 (6.8%)	12/238 (5.0%)	25/144 (17.4%)	5/25 (20.0%)	1/20 (5.0%)	65/752 (8.6%)	

*Theileria* spp.	*T. equi*, *Theileria* sp. NG-2013a	*T. equi*, *Theileria* sp. OT3, *Theileria* sp. B15a, *B. vulpes, *or closely related organism	*T. equi*, *Theileria* sp. OT3, *B. vulpes, *or closely related organism	*T. equi*, *Theileria* sp. YW-2014	*T. equi*		

^*∗*^No specimen was available.

**Table 4:** The *Theileria *spp. identified in livestock from five Caribbean islands and their similarity with reported organisms on GenBank.

Sequences identified in this study			Highly similar sequences in GenBank		Mismatch
*Theileria* spp.	Number	Source	GenBank #	Source	
*T. equi*	14	8 cattle, 1 goat from Nevis; 1 cow from Dominica; 3 donkeys, 1 sheep from St. Kitts	KF559357	Horse from China	0/550

*Theileria* sp. OT3	11	8 sheep, 3 goats from Montserrat	KF470868	Sheep from China	0/555

*Theileria* sp. NG-2013a	7	6 cattle from Nevis; 1 cow from Dominica	KF597076	Waterbuck from Kenya	0/552

*Theileria* sp. YW-2014	1	1 donkey from St. Kitts	AB981984	Sika deer from Japan	0/549

*Theileria* sp. B15a	1	1 goat from Grenada	JN572700	Buffalo from South Africa	0/539

*B. vulpes *or closely related organism	9	4 sheep, 4 goats from Montserrat; 1 goat from Dominica	JX454779	Dog from France	0/178
